# Genotypes of Bovine Viral Diarrhea Virus Infecting Vaccinated and Nonvaccinated Cattle in Thrace District, Türkiye

**DOI:** 10.3390/v18080807

**Published:** 2026-07-23

**Authors:** Gizem Karadag, Hasan Emre Tali, Ismail Egemen Ozkan, Nuri Turan, Sajid Umar, Juergen A. Richt, Huseyin Yilmaz, Aysun Yilmaz

**Affiliations:** 1 Department of Virology, Veterinary Faculty, Istanbul University-Cerrahpasa, Hadimkoy, Buyukcekmece, Istanbul 34500, Türkiye; gizem.akbaba@tarimorman.gov.tr (G.K.); hasan.tali@iuc.edu.tr (H.E.T.); ismail.ozkan@iuc.edu.tr (I.E.O.); nturan@iuc.edu.tr (N.T.); hyilmaz@iuc.edu.tr (H.Y.); 2 Division of Natural & Applied Sciences (DNAS), Duke Kunshan University, Kunshan 215316, China; sajid.umar@dukekunshan.edu.cn; 3 Global Health Research Center (GHRC), Duke Kunshan University, Kunshan 215316, China; 4 Department of Diagnostic Medicine and Pathobiology, College of Veterinary Medicine, Kansas State University, Manhattan, KS 66506, USA; jricht@vet.k-state.edu; 5 Department of Veterinary Tropical Diseases, Faculty of Veterinary Science, University of Pretoria, Onderstepoort 0110, Hatfield, Pretoria 0028, South Africa

**Keywords:** bovine viral diarrhea virus, cattle, genotypes, phylogenetics, Türkiye

## Abstract

Bovine viral diarrhea virus (BVDV) is a major cause of economic losses in the global cattle industry. This study investigated the clinical involvement and genetic diversity of BVDV in clinically affected cattle from the Thrace region in Türkiye, a critical border area with the European Union. A total of 533 nasal and rectal swabs were collected from 26 farms exhibiting clinical respiratory disease or diarrhea and analyzed using real-time RT-PCR. Positive samples were further characterized by sequencing and phylogenetic analysis to determine viral genotypes and subgenotypes. BVDV RNA was detected in 9 out of 26 farms (34.6%), with an overall positivity rate of 17.8% (95/533). Statistical analysis revealed significant associations between PCR positivity and both sampling year (*p* < 0.05) and animal age (*p* < 0.05). Genetic analysis of partial 5′-UTR sequences identified nine *Pestivirus bovis* subgenotype 1a strains, one *Pestivirus bovis* subgenotype 1f, and one *Pestivirus brazilense*. The *Pestivirus bovis* 1a strains clustered distinctly from previously reported Turkish BVDV-1 isolates, showing 97.32–99.11% nucleotide similarity with strains from Türkiye, Germany, China, Japan, the USA, Iran, and Argentina. Among the 533 samples tested, one Pestivirus brazilense isolate was identified, indicating its rare occurrence in the studied population despite targeted screening. This study represents the first report of *Pestivirus brazilense* in the Thrace region of Türkiye, expanding its known geographic range. The epidemiological data and genetic diversity analysis of BVDV strains characterized in this study offer critical insights for refining BVDV control programs, informing regional risk assessment, and supporting vaccine development.

## 1. Introduction

Bovine viral diarrhea virus (BVDV) is a major viral pathogen contributing to respiratory disease complexes in cattle and other ruminants. With its global prevalence, BVDV poses a significant threat to the cattle industry. BVDV naturally infects a wide range of ruminants and other artiodactyls, including cattle, yaks, buffalo, sheep, goats, deer, elk, antelope, and pigs, as well as camelids. Infections cause significant economic losses worldwide, including in Türkiye [1,2,3,4,5,6,7]. Amongst these animals, cattle are frequently infected with BVDV resulting in acute and persistent infections, mucosal disease, immunosuppression, enteric, respiratory system related and reproductive disorders such as leucopenia, fever, diarrhea, infertility, abortion, stillbirth, abnormal fetuses and decrease in milk and meat production [5,6,7,8]. The World Organization for Animal Health (WOAH) lists BVD as a notifiable disease due to its significant impact on animal health and welfare [6].

BVDV belongs to the *Pestivirus* genus within the *Flaviviridae* family [9,10]. The BVDV genome is a single-stranded, positive-sense RNA molecule of approximately 12.3–12.5 kb, consisting of a single open reading frame (ORF) flanked by 5′ and 3′ untranslated regions (UTRs). The ORF encodes a large polyprotein that is cleaved by viral and host proteases to produce four structural proteins (Capsid, Erns, E1, E2) and eight nonstructural proteins (Npro, p7, NS2, NS3, NS4A, NS4B, NS5A, NS5B) [10,11]. Based on their cytopathogenicity in cell culture, BVDV strains are classified into two biotypes: cytopathic (CP) and noncytopathic (NCP) [12]. Although both biotypes are pathogenic for cattle, the majority of persistent infections (PI) occur due to NCP strains during gestation of cows [6,13].

BVDV comprises three species: *Pestivirus bovis* (formerly BVDV-1), *Pestivirus tauri* (formerly BVDV-2), and *Pestivirus brazilense* (formerly HoBi-like pestivirus) [9,14,15,16,17,18]. To date, 24 *Pestivirus bovis* sub-genotypes (BVDV-1a to 1x) have been identified, while *Pestivirus tauri* and *Pestivirus brazilense* each have five (BVDV-2a to -2e and a–e, respectively) [2,19,20]. *Pestivirus brazilense* is also referred to by its geographic origin, such as Thai, Brazilian, or Italian strains [16,19,20,21,22].

BVDV transmission occurs through both vertical (transplacental) and horizontal routes, including direct contact, fomites, and reproductive fluid [23]. Calves born to BVDV infected cows can be persistently infected and continuously shed the virus [24,25]. BVDV can also be spread by acutely infected animals that shed the virus before developing an immune response to the virus [25]. BVDV can be introduced into herds in regions that are not infected with BVDV through a variety of routes, including the trade in live cattle, cross-border movements of domestic and wild animal populations, and contaminated fomites as well as artificial insemination and, less likely, transmission by vectors [26,27,28,29,30].

The expansion of international trade in live animals and animal products (including serum, embryos, and semen) has made rapidly developing livestock industries particularly vulnerable to BVDV transmission. This is reflected in the disease’s global seroprevalence, which currently exceeds 50% in many regions [17]. Türkiye maintains a substantial cattle population, with approximately 16 million head reported in 2023 [31]. Türkiye’s cattle production combines large integrated farms with small-scale family holdings. Integrated farms maintain hundreds of animals with standardized management, biosecurity, and vaccination programs, while small family farms typically keep fewer than 50 animals with limited biosecurity, infrequent vaccination, and traditional grazing practices, including communal pasture use. Over the past decade, intensified domestic and international cattle movement and trade of bovine products have significantly elevated the risk of BVDV transmission at both national and global levels [32].

Globally, *Pestivirus bovis* subgenotypes 1a–1u, along with novel variants 1v and 1w, as well as *Pestivirus tauri* subgenotypes 2a–2d and *Pestivirus brazilense*, have been reported in cattle populations [21,33,34,35]. In Europe, including Italy, Germany, Poland, Slovenia, and the UK, five to fifteen BVDV subgenotypes have been documented, with 1a, 1b, 1d, 1e, and 1f being predominant [36,37,38,39,40]. The United States primarily reports 1a, 1b, and *Pestivirus tauri* 2a [41], while Japan and Korea are dominated by 1b [42,43]. China has reported multiple *Pestivirus bovis* and *Pestivirus tauri* subgenotypes, including *Pestivirus brazilense* [4,6,44,45]. These findings highlight the global circulation of diverse *Pestivirus* genotypes and subgenotypes.

Compounding this risk, the lack of standardized national BVDV control and eradication programs has facilitated viral dissemination, particularly among smallholder farms in rural communities. Currently, there is scarcity of epidemiological investigations on BVDV in Türkiye. Comprehensive assessment of BVDV seroprevalence, and viral genetic diversity is essential for developing effective control strategies, including infection source identification, vaccine development, and immunization protocols. Notably, despite its strategic location bordering the European Union, the Thrace region lacks contemporary epidemiological data on BVDV. This study was therefore conducted to: (1) determine BVDV positivity rate in Thrace, Türkiye; (2) characterize circulating strain diversity; and (3) identify potential emerging variants.

## 2. Materials and Methods

### 2.1. Ethical Statement

This study involved non-invasive sample collection, limited to swabs, with no invasive procedures such as blood draws. All samples were obtained by registered veterinarians in accordance with good clinical practices and animal welfare standards. The collection process adhered to relevant national, international, and institutional guidelines for the ethical treatment and use of animals.

### 2.2. Study Population and Sampling

The study was conducted in cattle farms located in the Thrace region, the European part of Türkiye, which borders Bulgaria and Greece. A targeted sampling strategy was employed, selecting only animals with clinical signs of respiratory disease (dyspnea, coughing, nasal discharge, pneumonia) or diarrhea. This approach was chosen to assess the potential role of BVDV in clinical disease presentations, but it introduces selection bias and limits the generalizability of detection rate estimates to the broader cattle population. Clinically healthy cattle that did not exhibit any symptoms were excluded from sampling. A total of 26 herds, 20 integrated farms and 6 small family-owned farms were randomly selected from a list of herds reporting clinical respiratory disease or diarrhea. Sampling was carried out between September 2021 and January 2023, covering the autumn and winter seasons. The study included calves under 12 months of age and cattle aged 1–10 years exhibiting respiratory disease or diarrhea. Detailed farm data, clinical signs, and animal signalments are provided in the Supplementary Materials (see Supplementary Table S1). A total of 533 animals were sampled: 450 from integrated farms and 83 from small family-owned farms across villages in the provinces of Edirne, Kırklareli, Tekirdağ, and Istanbul. Of these, 345 animals had been vaccinated with a commercial inactivated BVDV vaccine containing BVDV-1 strain. Nasal swabs (*n* = 259) and/or rectal swabs (*n* = 274) were collected from each animal (see Supplementary Table S1). To identify potentially persistently infected (PI) animals, follow-up sampling would have been required; however, due to logistical constraints, this was not performed in the current study. Consequently, the positivity rate estimates reported herein reflect BVDV RNA detection at a single time point and may include both acute and persistent infections.

### 2.3. Extraction of RNA and cDNA Synthesis

Viral RNA was extracted from 200 μL of nasal and rectal swab transport medium (containing the swab sample after vortexing) using the PureLink™ RNA Mini Kit (Invitrogen, Cat. No: 12183020, Austin, TX, USA) following the manufacturer’s protocol. RNA was eluted in 50 μL of RNase-free elution buffer provided with the kit. RNA concentration and purity were assessed using a NanoDrop 1000c spectrophotometer (Thermo Scientific, Waltham, MA, USA). RNA concentration and purity were assessed using a NanoDrop 1000c spectrophotometer (Thermo Scientific, USA). To evaluate potential PCR inhibitors, each RNA sample was spiked with a known quantity of an exogenous internal control RNA (MS2 phage RNA, Roche, Cat. No: 10165948001) prior to cDNA synthesis. The internal control was amplified using specific primers and probe in a separate real-time RT-PCR reaction, as previously described by Hoffmann et al. [46]. Samples showing inhibition (i.e., >2 Ct delay compared to the internal control positive control) were diluted 1:10 with nuclease-free water and re-analyzed. All samples included in this study showed no significant inhibition after dilution. Complementary DNA (cDNA) was synthesized from 10 μL of extracted RNA (from the 50 μL eluate) using the High-Capacity cDNA Reverse Transcription Kit (Applied Biosystems, Cat. No: 4368814, Foster City, CA, USA) in a 20 μL total reaction volume, according to the manufacturer’s instructions. The resulting cDNA was stored at −80 °C until further use.

### 2.4. Initial Screening for BVDV-RNA by Real Time RT-PCR

For BVDV detection, primers and a TaqMan probe targeting the 5′NTR gene (206 bp) of Pestivirus were used as previously described by Hoffmann et al. [46]. The sequences of the primers and probe were: forward primer BVDV-Q-F1 (5′-CCTGAGTACAGGGTAGTCGTCAGT-3′), reverse primer BVDV-Q-R2 (5′-CAACTCCATGTGCCATGTACAG-3′), and TaqMan probe BVDV-Q-P1 (5′-FAM-TGCCCAGCCAGACTGAAGGGTGC-TAMRA-3′). An optimized 25 μL PCR reaction consisted of 3 μL cDNA, 1.5 μL of forward and reverse primers (final concentration: 0.6 μM), 0.5 μL of TaqMan probe (final concentration: 0.2 μM), 12.5 μL FluoCycle II Master Mix for Probe (Euroclone, Kat. No: ERD001100BIM, Milan, Italy) and 6 μL nuclease free water. A positive control, derived from BVDV-confirmed samples (obtained from the Department of Virology, Veterinary Faculty of Istanbul), was included in all PCR runs. Nuclease-free water served as the negative control, replacing the template. Amplifications were carried out in a real-time PCR instrument (Thermo Fisher Scientific, Applied Biosystems, StepOnePlus, Waltham, MA, USA) under the following cycling conditions: 95 °C for 10 min followed by 42 cycles of 94 °C for 30 s, 57 °C for 45 s, 68 °C for 45 s and final extension at 72 °C for 5 min. The real time RT-PCR control was performed by using beta-actin gene, as described previously [46]. Beta-actin amplification was conducted as an independent single-phase assay (not in duplex format with the BVDV screening reaction) using the following primers: beta-actin-F (5′-CACCACAGCTGAGAGGGAAATC-3′) and beta-actin-R (5′-GATCCACGACGGAGCATGG-3′), with a TaqMan probe beta-actin-P (5′-FAM-ACAGCCCTGGTCCCTGGTGGT-TAMRA-3′). The beta-actin reaction was performed in a 25 μL volume containing 3 μL cDNA, 1.5 μL of each primer (final concentration: 0.6 μM), 0.5 μL of TaqMan probe (final concentration: 0.2 μM), 12.5 μL FluoCycle II Master Mix for Probe (Euroclone, Cat. No: ERD001100BIM, Milan, Italy), and 6 μL nuclease-free water. Amplification conditions were: 95 °C for 10 min, followed by 40 cycles of 95 °C for 15 s and 60 °C for 60 s. All samples with beta-actin Ct values > 32 were considered to have insufficient RNA quality and were excluded from the analysis.

### 2.5. RT-PCR for Sequencing

Samples that tested positive by real-time RT-PCR were further analyzed using conventional RT-PCR to amplify a partial region of the 5′-UTR gene of BVDV, following previously published primers and methods described by Peddireddi et al. [47]. For amplification of the partial 5′-UTR gene of BVDV (293 bp), we used the following primers: BVDV-190F (5′-CCTGAGTACAGGGTAGTCGTCAGT-3′) and BVDV-324R (5′-TGCTATGAACACTCTACGAGAAC-3′). An optimized 25 μL RT-PCR reaction was composed of 4 μL of cDNA, 1 μL (0.4 μM) forward and reverse primers, 12.5 µL Platinum™ Hot Start PCR Master Mix (2X) (Invitrogen™, Kat. No: 13000012, Waltham, MA, USA), and 6.5 μL nuclease free water. Positive and negative controls were included in all reactions, as described in the real-time RT-PCR protocol. Amplifications were performed in a PCR instrument (Thermo Fisher Scientific, Applied Biosystems, StepOnePlus, Waltham, MA, USA) under the following cycling conditions: 95 °C for 10 min followed by 40 cycles of 95 °C for 20 s, 58 °C for 30 s and 72 °C for 45 s. PCR amplicons were electrophoresed on a 1.5% agarose gel, and a ~293 bp product was visualized using a gel imaging analysis system (Analytik Jena, Jena, Germany). The amplified products were then purified and sent for Sanger sequencing (MedSantek, Istanbul, Türkiye). For the identification of BVDV-3 (Hobi-like pestiviruses), the following primers were used to amplify partial 5′-UTR gene (150 bp): Hobi-F (5′-GCTAGCCATGCTCTTAGGAG-3′) and Hobi-R (5′-TGTGTCCATCAACCATAAACTC-3′), following the protocol described by Monteiro et al. [48]. An optimized 25 μL RT-PCR reaction was composed of 4 μL of cDNA, 1.25 μL (0.5 μM) forward and reverse primers, 12.5 µL Platinum™ Hot Start PCR Master Mix (2X) (Invitrogen™, Kat. No: 13000012, Waltham, MA, USA), and 6 μL nuclease free water. For all PCR reactions, positive and negative controls were kept as explained above for real time RT-PCR. All amplifications were performed in a PCR instrument (Thermo Fisher Scientific, Applied Biosystems, StepOnePlus, Waltham, MA, USA) by using the cycling conditions as follows: at 95 °C for 10 min followed by 35 cycles of 95 °C for 30 s, 55 °C for 30 s, 72 °C for 30 s and final extension at 72 °C for 5 min. Amplified PCR products (150 bp) were sent to a commercial company for sequence analysis (MedSantek, Istanbul, Türkiye).

### 2.6. Phylogenetic Analysis

The partial 5′-UTR gene sequences (293 bp) of BVDV obtained in this study were edited and verified using SnapGene (v7.2.1) and Chromas Pro (v2.1.10.1, Technelysium). For comparative genotyping, these sequences were aligned with reference BVDV 5′-UTR sequences from the NCBI database using MAFFT (v7) online alignment tool (https://mafft.cbrc.jp/alignment/server/ accessed on 21 June 2025). For the *Pestivirus brazilense* isolate, a 150 bp sequence was included in the phylogenetic analysis after alignment with corresponding regions of reference sequences. A phylogenetic tree was created using the Maximum Likelihood (ML) method with 1000 bootstrap replicates in MEGA 11 [49]. Bootstrap support values are indicated on the phylogenetic tree, with values ≥ 70% considered statistically significant. The reference strains used for phylogenetic comparison are listed in Supplementary Table S2. It should be noted that phylogenetic analysis based solely on partial 5′-UTR sequences, while sufficient for genotype assignment, provides limited resolution for distinguishing closely related strains and cannot definitively establish epidemiological linkages or identify novel variants. Sequencing additional genomic regions (e.g., Npro, E2, or NS5B) would be required for more robust phylogenetic inference. To assess genetic relatedness, data was generated using MegAlign Pro and GraphPad Prism (v10.3.1), illustrating the percentage homology among BVDV strains. Eleven sequences comprising 10 BVDV-1 strains and 1 *Pestivirus brazilense* isolate were deposited in GenBank under accession numbers: PQ432872-PQ432882.

### 2.7. Statistical Analyses

Statistical analyses were conducted using GraphPad Prism software (Version 10.3.1). Fisher’s exact test [50] was employed to compare sample types, demographic data, vaccination status, clinical signs in animals, and real-time RT-PCR results.

## 3. Results

### 3.1. Clinical Findings

Among the 533 animals studied, the most common clinical signs were diarrhea and respiratory symptoms (Supplementary Table S1). Of these, 258 exhibited respiratory signs such as wheezing, dyspnea, labored breathing, or increased respiratory rate while 156 presented with diarrhea alone. Additionally, 119 animals displayed both diarrhea and respiratory symptoms. Detailed information on sample identity, type, collection year, geographic origin, detected BVDV genotype, Ct values of positive samples, animal age, vaccination status, and GenBank accession numbers for BVDV sequences are provided in Supplementary Table S2. Notably, 345 out of the 533 animals had been vaccinated with an inactivated BVDV vaccine. Based on information provided by the farm managers, the inactivated vaccine contained a BVDV-1 strain; however, the specific subgenotype (e.g., 1a, 1b) and manufacturer details could not be reliably verified for all farms, and this represents a limitation of our study.

### 3.2. Overall Detection of Pestivirus-RNA

**Figure 1:**
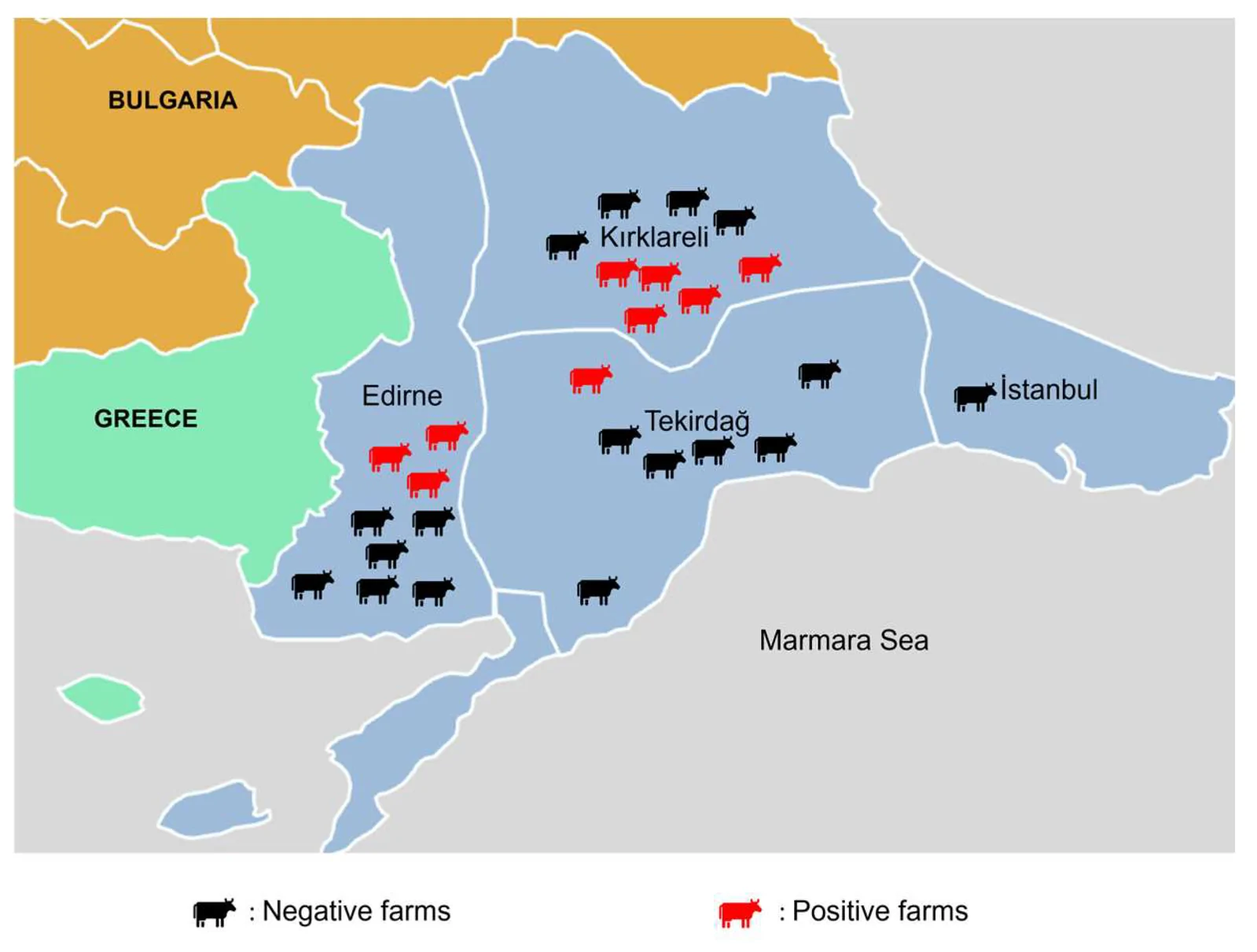
The sampling area (EU border), number of farms analyzed and number of BVDV-RNA positive farms analyzed in this study.

Among the 26 farms sampled, nine (34.6%) tested positive for BVDV-RNA via real-time RT-PCR (Figure 1, Supplementary Figure S1). Of these, five were small family-owned farms, and four were integrated farms, though the difference was not statistically significant (Table 1). BVDV-RNA was detected in 5% of 221 animals from 25 farms in 2021, 28% of 214 animals from two farms in 2022, and 26% of animals from three farms in 2023, with statistically significant variation (Table 1 and Table 2). Overall, 17.8% (95/533) of samples tested positive (Supplementary Figure S1). In vaccinated animals, Ct values ranged from 26.06 to 37.67, compared to 17.66–37.94 in non-vaccinated animals. Positivity rates were similar between nasal (17.4%, 45/259) and rectal swabs (18.2%, 50/274), with no statistical significance (Table 1). Of the BVDV-RNA-positive nasal swabs, 38 originated from farms in Kırklareli, six from Edirne, and one from Tekirdağ, with none from Istanbul. Thirty-one positive nasal swabs came from integrated farms, and 14 from family-owned farms. All positive rectal swabs were from Kırklareli, with 45 from integrated farms and five from family-owned farms. Provincial positivity rates were 6% (Tekirdağ), 16% (Edirne), and 19% (Kırklareli), with no statistically significant differences (Table 1). Among RT-PCR-positive animals, 23 exhibited both diarrhea and respiratory signs, while others showed either symptom alone. Dyspnea was observed in 60% of BVDV-RNA-positive animals, compared to 54% without dyspnea. Similarly, 53% of diarrheic animals tested positive versus 51% of non-diarrheic animals. No significant association was found between clinical signs and BVDV-RNA positivity (Supplementary Table S1).

Positivity rates varied significantly by age group: ≤3 months (8%), 3–6 months (30%), 6–12 months (26%), 1–2 years (22%), and >2 years (29%) (Table 1). This pattern coincides with the period of maternal antibody decline, which typically occurs between 2 and 6 months of age in calves. As maternal antibodies wane, calves become increasingly susceptible to BVDV infection through horizontal transmission from persistently infected herdmates or acutely infected animals. Vaccinated animals had a slightly higher positivity rate (20%) than non-vaccinated animals (14%), though this difference was not statistically significant (Supplementary Figure S2; Table 1).

### 3.3. Viral Load Assessment by Ct Values

Among the 95 real-time RT-PCR-positive samples, 81 exhibited high Ct values (>30), indicating low viral RNA loads, while 14 had low Ct values (≤30), reflecting higher viral loads. In vaccinated animals, Ct values ranged from 26.06 to 37.67, compared to 17.66–37.94 in non-vaccinated animals. The presence of samples with low viral loads (Ct > 30) suggests that a substantial proportion of positive animals may represent acute infections with low-level shedding, rather than PI that typically yield lower Ct values. The Ct value distribution also highlights the limitations of sequencing from samples with high Ct values, as insufficient viral RNA prevents successful PCR amplification for sequencing.

### 3.4. Sequencing and Phylogenetic Analysis

**Figure 2:**
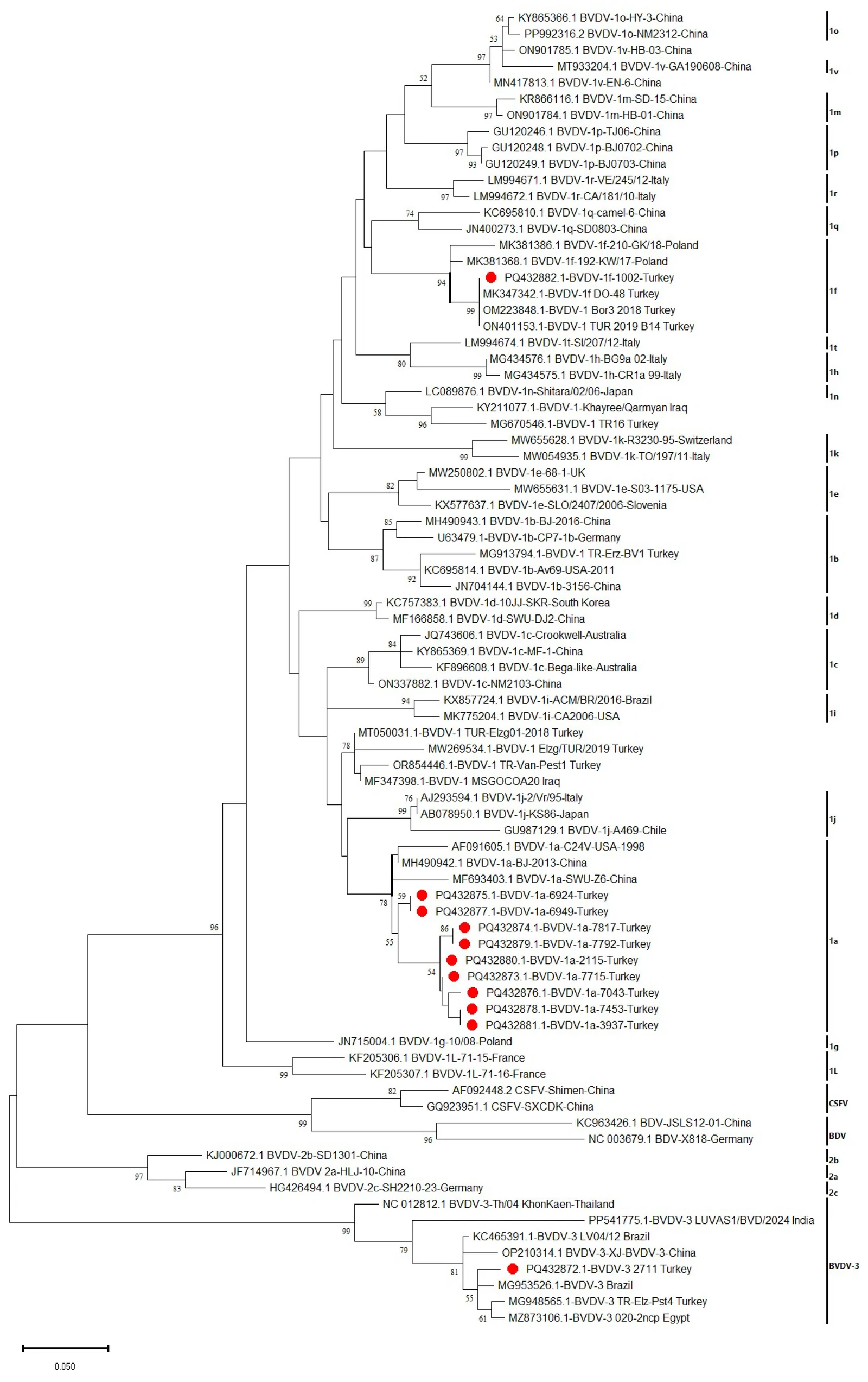
Phylogenetic tree generated using the sequences of 5′-UTR gene of BVDV submitted to GenBank. Figure shows the clusters of *Pestivirus bovis*, *Pestivirus tauri* and *Pestivirus brazilense*. Sequences with red round circles indicate the viruses found in this study (PQ432872, PQ432873, PQ432874, PQ432875 PQ432876, PQ432877, PQ432878, PQ432879, PQ432880, PQ432881, PQ432882).

Phylogenetic analysis revealed that 10 BVDV isolates clustered within BVDV-1, while one isolate grouped with *Pestivirus brazilense* (Figure 2). Among the BVDV-1-positive isolates, nine were classified as subgenotype *1a*, exhibiting 91.96–100% nucleotide homology with each other, and one was identified as *Pestivirus bovis* subgenotype *1f* (99.11% homology) (Table 2). Detailed metadata for these samples including sample type (nasal/rectal), Ct values, subgenotype, geographic origin, sampling dates, animal age, vaccination status, and GenBank accession numbers are provided in Supplementary Table S3. Despite the use of species-specific primers and a dedicated RT-PCR protocol for *Pestivirus brazilense* detection [48], only one sample (0.2%, 1/533) tested positive for this species. This finding suggests that *Pestivirus brazilense* remains rare in the Thrace region population.

The nine *Pestivirus bovis* subgenotype 1a strains from this study (PQ432873-PQ432881) exhibited 89.58–97.92% similarity with previously reported Turkish BVDV-1a strains (e.g., MT050031), with PQ432879 showing the highest similarity (97.92%). Phylogenetically, these strains clustered closely with BVDV 1a strains from China (MH490942, MF693403) and USA (AF091605) (Figure 2). Notably, the BVDV-1a subgenotypes identified in this study formed a distinct phylogenetic cluster separate from previously characterized BVDV-1 strains in Türkiye (Figure 2), suggesting potential genetic divergence or the emergence of novel variants. The single *Pestivirus bovis* subgenotype 1f strain (PQ432882) showed 93.75–98.96% similarity with Turkish isolates (e.g., OM223848), with the highest match (98.96%) to three local strains. Globally, it shared 89.58–93.75% similarity with European strains (Italy, Germany, Poland, Slovenia). The BVDV-1f isolate (PQ432882) exhibited the lowest Ct value (17.66) among all sequenced samples, indicating the highest viral RNA load. This animal, a 3-month-old calf from Kırklareli province, presented with severe diarrhea and respiratory distress. While this single observation suggests a potential association between BVDV-1f infection and more severe clinical presentation, the limited number of sequenced isolates (*n* = 11) precludes definitive conclusions about subgenotype-specific pathogenicity. Further studies with larger sample sizes and comprehensive clinical scoring systems are needed to investigate potential virulence differences among BVDV subgenotypes circulating in Türkiye. The detected *Pestivirus brazilense* strain (PQ432872) was 100% identical to a previously reported Turkish strain (MG948565). Detailed similarity percentages are summarized in Table 2, while sample metadata (Ct values, geographic origin, accession numbers, etc.) are provided in Supplementary Table S3.

## 4. Discussion

Endemic diseases impose substantial economic burdens on the Turkish beef and dairy industry. Among these, bovine viral diarrhea virus (BVDV) is particularly significant, affecting a large proportion of the cattle population across the country. Although Türkiye mandates BVDV screening for imported cattle, continuous surveillance remains essential to monitor the epidemiological status of this economically significant disease. This study provides an updated epidemiological snapshot of BVDV in the Thrace region of Türkiye, a critical border area with the European Union. While BVDV-1a, BVDV-1f, and *Pestivirus brazilense* have been previously reported in Türkiye [2,51,52,53], this is the first documentation of *Pestivirus brazilense* in the Thrace district, marking a significant expansion of its known geographic range within the country. Notably, *Pestivirus brazilense* is antigenically and genetically distinct from classical BVDV-1 and BVDV-2 strains, particularly in the E2 glycoprotein a major target of neutralizing antibodies. This antigenic divergence could potentially compromise vaccine efficacy, as neutralizing antibodies elicited by BVDV-1-based vaccines may provide limited protection against *Pestivirus brazilense* infection. The detection of co-circulating *Pestivirus bovis* subgenotypes 1a and 1f, alongside *Pestivirus brazilense*, demonstrates the growing genetic diversity of BVDV in a high-risk border region with Europe. The detection of *Pestivirus brazilense* in Thrace has significant implications for disease control in Türkiye. While *Pestivirus brazilense* (formerly HoBi-like pestivirus) has been associated with clinical syndromes similar to classical BVDV, including respiratory disease, diarrhea, and reproductive disorders, its relative pathogenicity compared to *Pestivirus bovis* and *Pestivirus tauri* remains incompletely understood. Notably, *Pestivirus brazilense* is antigenically and genetically distinct from classical BVDV-1 and BVDV-2 strains, particularly in the E2 glycoprotein—a major target of neutralizing antibodies. The E2 protein of *Pestivirus brazilense* shares approximately 70–75% amino acid identity with BVDV-1 reference strains, raising concerns about cross-protection from existing vaccines that predominantly contain BVDV-1 antigens. This antigenic divergence could potentially compromise vaccine efficacy, as neutralizing antibodies elicited by BVDV-1-based vaccines may provide limited protection against *Pestivirus brazilense* infection. Further studies are urgently needed to assess the cross-neutralizing activity of sera from vaccinated cattle against circulating *Pestivirus brazilense* strains in Türkiye and to evaluate whether vaccine updates or modified vaccination strategies are required to address this emerging threat. However, the targeted sampling of clinically affected animals and the reliance on partial 5′-UTR sequences represent important limitations that must be considered when interpreting these findings.

Antibodies against BVDV have been detected in both vaccinated and non-vaccinated cattle in Türkiye, with reported seroprevalence rates varying across studies [41,42,43,44,45,46,47,48]. These differences are influenced by factors such as geographic region, year of study, production system, vaccination status, and animal age [53,54,55,56]. Notably, the highest reported seroprevalence in non-vaccinated cattle reached 96.04% [57]. In the Aegean region of Türkiye, which shares similar livestock production characteristics with the Thrace region, İnce and Ayaz [56] reported that the herd-level and animal-level seroprevalence of BVDV was 89.58% (95% CI: 77.83–95.47) and 48.37% (95% CI: 44.23–52.54), respectively. However, this study was conducted approximately seven years ago, and the contemporary serological status of BVDV in the Aegean region remains poorly characterized. Although our study did not include serological testing, the detection of BVDV RNA in 17.8% of clinically affected animals in our study suggests active viral circulation in the Thrace region. Seroprevalence data would be valuable to assess the extent of past and current exposure in this specific region. A meta-analysis of 128 studies estimated the global seroprevalence of BVDV at 42.77% (95% CI: 37.01–48.63) [5]. In Türkiye, studies using real-time RT-PCR and conventional RT-PCR have reported variable detection rates, influenced by geographic region, vaccination status, and sample type. On average, a 5% positivity rate has been observed [2,58,59,60], with notably higher rates (up to 73.6%) detected in aborted fetuses [12]. In this study, BVDV-RNA was detected by real-time RT-PCR in 9 out of 26 sampled farms (34.6% herd level detection rate). While no statistically significant association was observed between PCR positivity and farm type, the highest detection rate occurred in Kırklareli province, suggesting potential regional epidemiological variation. Among 533 tested samples, 95 (17.8%) were BVDV-RNA positive a notably higher rate compared to both a recent meta-analysis of 318 studies (pooled detection rate: 0.08%) [38] and global surveillance data [39]. The lack of a significant difference in BVDV-RNA detection rate between diarrheic (53%) and non-diarrheic (51%) animals suggests that BVDV was not the sole causative agent of diarrhea in this cohort, and other pathogens or factors were likely involved in a substantial proportion of cases. Further analysis revealed no significant associations between PCR positivity and vaccination status, geographical location, or sample type.

Among RT-PCR-positive animals (*n* = 95), clinical presentations included: diarrhea alone (*n* = 33, 34.7%), respiratory signs alone (*n* = 39, 41.1%), and both diarrhea and respiratory signs (*n* = 23, 24.2%). No significant association was found between specific clinical signs and BVDV-RNA positivity. The lack of a significant difference in BVDV-RNA detection rate between diarrheic (53%) and non-diarrheic (51%) animals, and between animals with respiratory signs (54%) and without respiratory signs (60%), suggests that BVDV was not the sole causative agent of these clinical presentations. Other respiratory and enteric pathogens, including bovine respiratory syncytial virus, bovine parainfluenza virus-3, bovine coronavirus, and bacterial pathogens, were likely involved in a substantial proportion of cases. This pattern is consistent with the recognized role of BVDV as an immunosuppressive agent that predisposes cattle to secondary infections, rather than as a primary pathogen in all cases [61]. The detection of BVDV RNA in 17.8% of clinically affected animals in our study suggests active viral circulation in the Thrace region, but seroprevalence data would be valuable to assess the extent of past and current exposure in this specific region.

Persistently infected (PI) animals represent the primary reservoir for BVDV transmission within and between herds [2,33,51]. Calves born from BVDV-infected cows can be persistently infected and continuously shed the virus, contributing significantly to disease maintenance and spread [33,39]. The present study employed single-time-point sampling, which does not permit differentiation between transient acute infections and persistent infections. While the presence of animals with low Ct values (<30) in our study may suggest potential persistent infections, confirmation would require repeat sampling after 3–4 weeks to demonstrate sustained viremia. This limitation is particularly relevant given that PI animals are a key target for eradication programs and that their identification is critical for effective disease control [62,63]. Additionally, the targeted sampling strategy restricted to clinically affected animals likely underestimates the true prevalence in the general population, as subclinically infected animals were excluded from the study. Future studies should incorporate follow-up sampling to identify PI animals and employ random sampling strategies to generate unbiased prevalence estimates. In Türkiye, small family-owned farms typically do not vaccinate cattle against BVDV, making these animals potential key disseminators of the virus along with PI individuals.

The lack of significant difference in BVDV infection rates between cattle vaccinated with an inactivated vaccine and non-vaccinated cattle in our study should be interpreted with caution, as this observation may reflect multiple factors that cannot be disentangled from the current dataset. Potential explanations include: (1) antigenic mismatch between the vaccine strains and circulating field subgenotypes; (2) inadequate implementation of vaccination programs, including timing of administration relative to exposure; or (3) inherent differences in the efficacy of inactivated vaccines in preventing infection and viral shedding compared to modified-live virus vaccines [62,63,64]. Furthermore, many inactivated vaccines are designed primarily to prevent clinical disease; they may not prevent initial infection or short-term viral shedding (i.e., do not provide sterile immunity). However, without detailed information on vaccination protocols (including timing, number of doses, and vaccine strain) and without experimental challenge data, causal conclusions regarding vaccine effectiveness cannot be drawn. The higher positivity rate observed in vaccinated animals (20%) compared to non-vaccinated animals (14%) may also reflect confounding factors such as increased exposure risk in vaccinated herds or vaccination of already infected animals. The effectiveness of vaccination programs can be compromised by logistical delays, such as administering vaccines after animals have already been exposed. Furthermore, many inactivated vaccines are designed primarily to prevent clinical disease; they may not prevent initial infection or short-term viral shedding. These findings highlight the need for comprehensive investigations into vaccine efficacy and coverage in different farming systems, including controlled studies that account for confounding variables.

This study incorporated sampling across three consecutive years (2021–2023) and included animals of varying ages. Real-time PCR analysis revealed significantly lower BVDV-RNA detection rates in 2021 (5%) compared to subsequent years (2022: 28%; 2023: 26%) (*p* < 0.05), suggesting a potential temporal increase in detection or outbreak activity during the later years of the study. Notably, animals under 3 months of age showed the lowest positivity rates (8%), while significantly higher rates were observed in the 3–6 months (30%) and 6–12 months (26%) age groups (*p* < 0.0001). This pattern coincides with the period of maternal antibody decline, which typically occurs between 2 and 6 months of age in calves. As maternal antibodies wane, calves become increasingly susceptible to BVDV infection through horizontal transmission from persistently infected herdmates or acutely infected animals. The natural ‘booster’ effect from field infections in non-vaccinated herds might stimulate higher antibody titers than those provided by maternal antibodies or vaccination alone, potentially explaining the elevated positivity rates observed in older age groups. These findings align with global surveillance data [39] and support previous observations that older cattle develop immunity through either natural exposure or vaccination [4].

As an RNA virus, BVDV exhibits high genetic variability due to frequent mutations, leading to challenges in both diagnosis and vaccine efficacy. This genetic diversity results in varying distributions of BVDV genotypes and subgenotypes across different geographical regions [1,65,66]. Genotyping typically targets conserved regions including the 5′-UTR, E, NS5B, and NPro genes [2,67,68]. In this study, we focused on the 5′-UTR for phylogenetic analysis.

Previous studies in Türkiye have identified all three recognized BVDV species (*Pestivirus bovis*, *Pestivirus* tauri, and *Pestivirus* brazilense) through analysis of the 5′-UTR, Npro, and E genes, with *Pestiviru bovis* being the predominant species [2,51,60,69]. Notably, *Pestivirus brazilense* appears to be rare in Türkiye and other regions outside South America [34,48,70], possibly due to either limited diagnostic detection or insufficient investigation. To address this potential diagnostic gap, we employed specific primers and a dedicated RT-PCR protocol to enhance detection of *Pestivirus brazilense* [48], which identified only one positive sample (1/533, 0.2%).

Due to its superior sensitivity compared to conventional RT-PCR, real-time RT-PCR can detect even low viral loads. In this study, we identified 81 samples with high Ct values (>30), indicating the presence of BVDV RNA but at low concentrations. Despite using two different primer sets targeting *Pestivirus bovis*, *Pestivirus tauri*, and *Pestivirus brazilense*, we were unable to obtain sequences from these samples due to insufficient viral RNA copy numbers. In contrast, 11 of 14 samples with low Ct values (<30), reflecting higher viral loads, were successfully sequenced. We also attempted to sequence samples with intermediate Ct values (30–35), but no amplification was observed in the conventional RT-PCR step, preventing successful Sanger sequencing.

Phylogenetic analyses in this study revealed that 10 BVDV isolates clustered with *Pestivirus bovis*, while one sample grouped with *Pestivirus brazilense*. Among the *Pestivirus bovis*-positive samples, nine belonged to subgenotype BVDV-1a (showing 91.96–100% homology), and one was classified as subgenotype 1f (99.11% homology). Previous studies in Türkiye have identified *Pestivirus bovis* subgenotype 1l as the most prevalent, followed by 1f, while 1a, 1b, 1c, 1d, 1r, and 1v are detected at lower frequencies [33,51,58,60,67,69,70]. In contrast, 1a, 1b, 1d, 1e, and 1f dominate in European countries, whereas 1a, 1b, and *Pestivirus tauri* 2a are the most common in the United States [37,41,71]. Meanwhile, 1b is the predominant subgenotype in Japan and Korea, while 1c is more frequent in Australia [42,43,72]. China has reported multiple *Pestivirus bovis* and *Pestivirus tauri* subgenotypes, including *Pestivirus brazilense* [4,6]. These findings highlight the global circulation of diverse Pestivirus genotypes and subgenotypes.

The nine *Pestivirus bovis* subgenotype 1a strains identified in this study exhibited 89.58–97.92% similarity to previously reported Turkish 1a strains. Notably, they showed 100% similarity to a German reference strain (AF376001) and 97.92% similarity to strains from Italy, Slovakia, the USA, and Japan, while sharing 98.96% identity with Chinese and Uruguayan (KT833786) variants. However, the genetic relationships inferred from partial 5′-UTR sequences must be interpreted with caution. The 5′-UTR is a conserved region that provides limited phylogenetic resolution, and the observed high similarity does not conclusively establish epidemiological links or common origins. Full-genome sequencing or analysis of more variable regions (e.g., E2, NS5B) would be required to confirm these relationships with greater confidence. The 100% identity with a German reference strain suggests a possible epidemiological link or common source, though further analysis with full-genome sequences is needed to confirm this finding. The introduction of *Pestivirus brazilense* to Türkiye may have occurred through multiple routes: (1) importation of live cattle from South America, where *Pestivirus brazilense* is endemic and commonly detected in fetal bovine serum and biological products [73]; (2) during the importation of contaminated semen or embryos; or (3) through feed or biological products containing contaminated bovine serum. These findings suggest widespread circulation of closely related *Pestivirus bovis* 1a strains across Europe, Asia, the Americas, and the Middle East. The 100% match with the German strain and high similarity (98.96%) to Chinese and Uruguayan variants raise questions about a potential origin or epidemiological link to these regions.

The Ct value distribution observed in this study has important diagnostic and epidemiological implications. The 81 samples with high Ct values (>30) indicate low viral RNA loads, which may reflect acute infections with low-level virus shedding; late-stage infections with declining viral titers; or, in vaccinated animals, breakthrough infections with reduced viral replication. In contrast, the 14 samples with low Ct values (≤30) are more likely to represent active infections with high-level virus shedding, potentially including persistently infected animals [2,67]. The inability to sequence samples with high Ct values (despite using multiple primer sets) underscores the critical impact of viral load on sequencing success and highlights the limitations of molecular surveillance when relying solely on clinical samples. Future serological surveillance studies would be particularly valuable for differentiating vaccinated from naturally infected animals, as antibodies against non-structural protein 3 (NS3) are barely detectable in animals vaccinated with inactivated BVDV vaccines, whereas naturally infected or persistently infected animals develop robust NS3-specific antibody responses [74]. This distinction could help estimate the true infection rate in vaccinated populations and assess vaccine effectiveness in the field. Combined with virological surveillance, such serological approaches would provide a more comprehensive understanding of BVDV epidemiology in Türkiye.

While BVDV-1a, BVDV-1f, and *Pestivirus brazilense* have been previously reported elsewhere in Türkiye [2,51,58,59,67,70], this study provides three novel contributions: (1) first detection of *Pestivirus brazilense* in the Thrace region, a key entry point for cattle imports into Türkiye; (2) documentation of a shift in subgenotype predominance from previously reported BVDV-1f in Thrace [2] to co-circulation of BVDV-1a, BVDV-1f, and *Pestivirus brazilense*; and (3) updated epidemiological data for the 2021–2023 period, filling a critical gap in surveillance. The detection of emerging BVDV genotypes in Thrace, which serves as a critical epidemiological buffer between Turkey and the European Union, introduces substantial challenges for regional disease control. The potential of these novel variants to evade vaccine-induced immunity not only compromises the effectiveness of existing vaccination protocols but also elevates the risk of transboundary spread into EU member states. Consequently, significant financial and logistical resources are required to sustain intensive surveillance, enforce animal movement controls, and uphold the region’s essential disease-free certification.

Several limitations of this study should be acknowledged. First, the targeted sampling of only clinically affected animals introduces selection bias and limits the generalizability of detection rate estimates to the broader cattle population. Second, the absence of follow-up sampling precludes the identification of persistently infected animals, which are critical to the epidemiology and maintenance of BVDV within herds. Third, phylogenetic analysis was based solely on partial 5′-UTR sequences (293 bp for BVDV-1 and 150 bp for *Pestivirus brazilense*), which, while sufficient for genotype assignment, provides limited resolution for distinguishing closely related strains and cannot definitively establish epidemiological linkages or identify novel variants. Sequencing additional genomic regions (e.g., Npro, E2, or NS5B) would be required for more robust phylogenetic inference. Fourth, the lack of detailed vaccination protocol information limits the interpretability of comparisons between vaccinated and non-vaccinated animals. Fifth, the small number of sequenced samples (*n* = 11) constrains the robustness of molecular epidemiological conclusions. Future studies addressing these limitations through random sampling, longitudinal follow-up, and multi-gene sequencing would substantially enhance our understanding of BVDV epidemiology in Türkiye.

## 5. Conclusions

This study reports the first detection of *Pestivirus brazilense* in Türkiye’s Thrace district, marking a significant expansion of its known geographic range. Together with the identification of co-circulating *Pestivirus bovis* subgenotypes (including 1a and 1f), these results demonstrate the growing genetic diversity of BVDV in Türkiye, particularly in a high-risk border region with Europe. This study provides an updated epidemiological profile of BVDV in Türkiye, identifying the predominant subgenotypes circulating over the past three years. By focusing on the Thrace district, a critical border region with Europe, we address a key gap in the molecular epidemiology of BVDV in Türkiye. Furthermore, the limitations of our study including targeted sampling of clinically affected animals, lack of PI animal identification, and reliance on partial 5′-UTR sequences should be considered when interpreting these findings. Our findings enhance understanding of viral diversity and genotype distribution, offering insights into potential epidemic trends and control measures. To optimize BVDV management, further epidemiological studies are urgently needed to assess BVDV positivity rate in Turkish herds using random sampling strategies and longitudinal follow-up to identify PI animals. Such data are vital for refining region-specific control policies, including targeted vaccination, biosecurity measures, and PI eradication programs.

## Figures and Tables

**Table 1:** Association between demographic and clinical variables and BVDV-RNA positivity by real-time RT-PCR: univariate analysis with odds ratios and 95% confidence intervals.

Variables	Level	Number of Animals	Real Time RT-PCR Negative	Real Time RT-PCR Positive	Positive %	Logistic Regression	
						OR (95%CI)	*p* Value
Overall positivity	All animals	533	438	95	17.8	**-**	**-**
Year	2021	221	211	10	5	1 (ref)	**-**
	2022	214	154	60	28	8.221 (4.255–17.53)	<0.0001
	2023	98	73	25	26	7.226 (3.405–16.46)	
Locality	Istanbul	7	7	0	0	-	-
	Edirne	38	32	6	16	0.68 (0.47–0.98)	0.4490
	Kırklareli	471	383	88	19	0.79 (0.68–0.91)	
	Tekirdağ	17	16	1	6	0.20 (0.01–1.69)	
Farm type	Small family-owned farms	83	64	19	23	1 (ref)	-
	Integrated farms	450	374	76	17	1.45 (0.82–2.55)	0.2114
Age	1- (≤3 months)	264	244	20	8	1 (ref)	**-**
	2- (>3–≤6 months)	143	100	43	30	5.246 (2.976–9.530)	<0.0001
	3- (>6–≤12 months)	42	31	11	26	4.329 (1.851–9.775)	0.0005
	4- (>1–≤2 years)	46	36	10	22	3.389 (1.422–7.690)	0.0042
	5- (>2 years)	38	27	11	29	4.970 (2.105–11.37)	0.0002
Sample type	Rectal swabs	274	224	50	18	1 (ref)	-
	Nasal swabs	259	214	45	17	1.07 (0.68–1.67)	0.8215
Vaccination status	Vaccinated	345	277	68	20	1 (ref)	-
	Nonvaccinated	188	161	27	14	1.47 (0.90–2.41)	0.1549

**Table 2:** Percentage Similarity of 5′-UTR Gene Sequences Among BVDV Strains Detected in This Study.

Strain	PQ432872	PQ432873	PQ432874	PQ432875	PQ432876	PQ432877	PQ432878	PQ432879	PQ432880	PQ432881	PQ432882
PQ432872 (BVDV-3)	100	-	-	-	-	-	-	-	-	-	-
PQ432873 (BVDV-1a)	91.96	100	-	-	-	-	-	-	-	-	-
PQ432874 (BVDV-1a)	91.96	100	100	-	-	-	-	-	-	-	-
PQ432875 (BVDV-1a)	91.96	100	100	100	-	-	-	-	-	-	-
PQ432876 (BVDV-1a)	92.86	96.43	96.43	96.43	100	-	-	-	-	-	-
PQ432877 (BVDV-1a)	92.86	96.43	96.43	96.43	100	100	-	-	-	-	-
PQ432878 (BVDV-1a)	93.75	95.54	95.54	95.54	94.64	94.64	100	-	-	-	-
PQ432879 (BVDV-1a)	93.75	95.54	95.54	95.54	94.64	94.64	100	100	-	-	-
PQ432880 (BVDV-1a)	92.86	96.43	96.43	96.43	100	100	94.64	94.64	100	-	-
PQ432881 (BVDV-1a)	92.86	96.43	96.43	96.43	100	100	94.64	94.64	100	100	-
PQ432882 (BVDV-1f)	93.75	95.54	95.54	95.54	98.21	98.21	96.43	96.43	98.21	98.21	100

## Data Availability

Data are available upon reasonable request from the corresponding authors.

## Supplementary Materials

The following supporting information can be downloaded at: https://www.mdpi.com/article/10.3390/v18080807/s1, Supplementary Figure S1. Number of farms, farm type and number of positive samples analyzed in this study. E: Integrated farms; F: Small family-owned farms. Supplementary Figure S2. Number of farms, sampling years, vaccination status, number of positives and negatives in integrated and small family-owned farms. Supplementary Table S1. Data about farms, clinical signs and signalments of animals analyzed in this study. Supplementary Table S2. List of reference strains for phylogenetic comparison with isolates Supplementary Table S3. Sample identity, sample type, sampling year, sampled city, BVDV genotype detected, Ct values of positive samples, age and vaccination status of animals and GenBank accession numbers of BVDV sequences obtained in this study.
