# Supplementary material for: Genotypes of Bovine Viral Diarrhea Virus Infecting Vaccinated and Nonvaccinated Cattle in Thrace District, Türkiye
Source: Viruses. 2026 Jul 23;18(8):807. doi: 10.3390/v18080807 (PMC13517928; doi:10.3390/v18080807)
Supplement: Supplementary file 1 [file viruses-18-00807-s001.zip › Supplementary Figure S1.pdf]

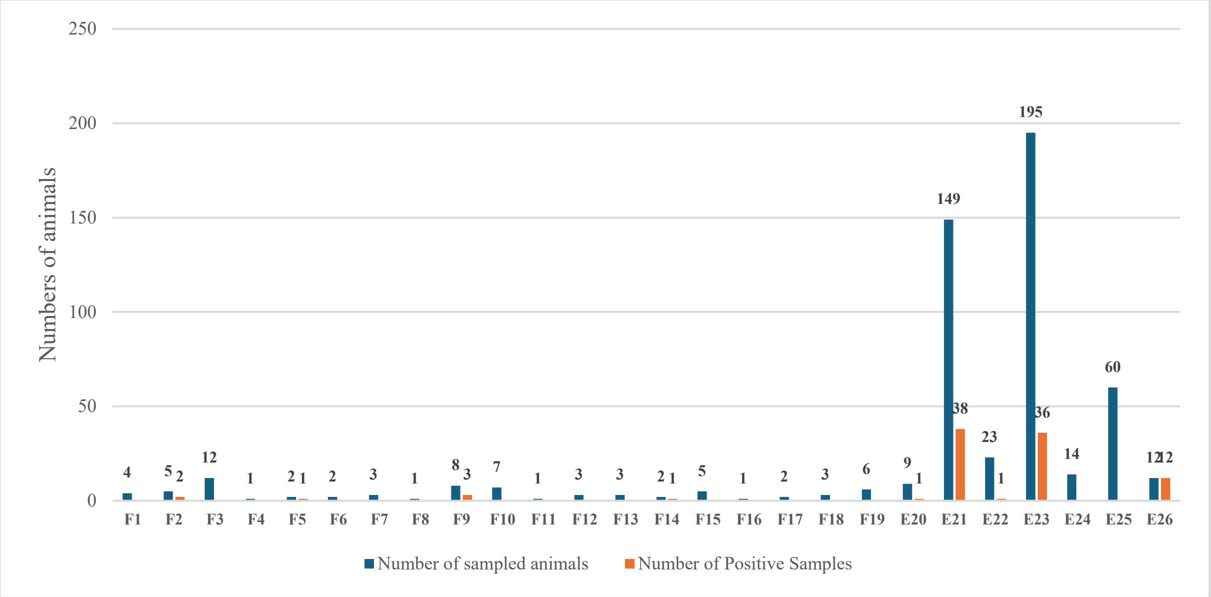

Supplementary Figure S1. Number of farms, farm type and number of positive samples analysed in this study. **E:** Integrated farms; **F:** Small family-owned farms.
