# Supplementary material for: Genotypes of Bovine Viral Diarrhea Virus Infecting Vaccinated and Nonvaccinated Cattle in Thrace District, Türkiye
Source: Viruses. 2026 Jul 23;18(8):807. doi: 10.3390/v18080807 (PMC13517928; doi:10.3390/v18080807)
Supplement: Supplementary file 1 [file viruses-18-00807-s001.zip › Supplementary Figure S2.pdf]

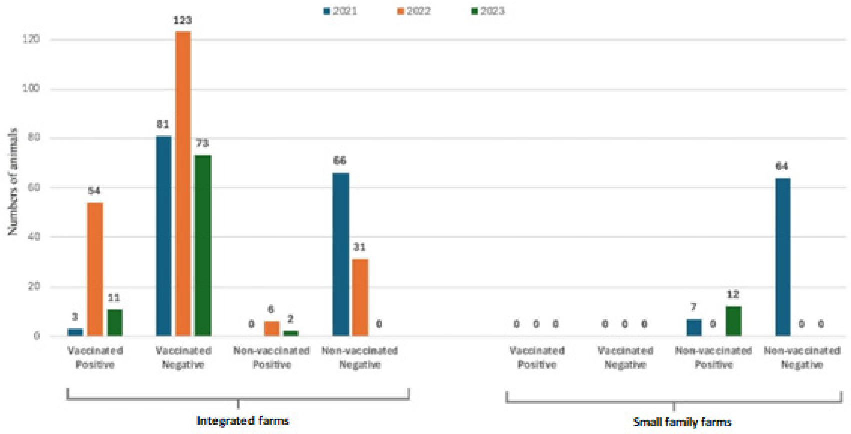

Supplementary Figure S2. Number of farms, sampling years, vaccination status, number of positives and negatives in integrated and small family-owned farms.
