# Supplementary material for: Genotypes of Bovine Viral Diarrhea Virus Infecting Vaccinated and Nonvaccinated Cattle in Thrace District, Türkiye
Source: Viruses. 2026 Jul 23;18(8):807. doi: 10.3390/v18080807 (PMC13517928; doi:10.3390/v18080807)
Supplement: Supplementary file 1 [file viruses-18-00807-s001.zip › Supplementary Table S1.pdf]

Supplementary Table S1. Data about farms, clinical signs and signalments of animals analyzed in this study.

								Sample type		Clinical signs					
Sampling year	Sampling city	Farm No	Age	Farm type	Production Type	Vaccination status	Number of sampled animals	rectal swab	Nasal swab	Dyspnea	Nasal disscharge	Coughing	Weakness	Fever	Diarrhea
2021	Edirne	1	0-3 Months	Small family owned	N/A	0	4	0	4	0	3	4	3	0	0
2021	Edirne	2	0-3 Months	Small family owned	Dairy	0	1	0	1	0	1	1	1	0	0
2021	Edirne	3	0-3 Months	Small family owned	N/A	0	12	0	12	0	12	5	12	7	0
2021	Edirne	4	0-3 Months	Small family owned	N/A	0	1	0	1	0	1	1	1	0	0
2021	Edirne	5	0-3 Months	Small family owned	Dairy	0	1	0	1	0	1	1	1	0	0
2021	Edirne	6	0-3 Months	Small family owned	N/A	0	1	0	1	0	1	1	1	0	0
2021	Edirne	7	0-3 Months	Small family owned	N/A	0	3	0	3	0	3	3	3	0	0
2021	Edirne	8	0-3 Months	Small family owned	N/A	0	1	0	1	0	1	1	1	0	0
2021	Edirne	6	3-6 Months	Small family owned	N/A	0	1	0	1	0	1	1	1	0	0
2021	Edirne	9	3-6 Months	Small family owned	Meat	0	6	0	6	1	6	6	6	0	0
2021	Edirne	9	3-6 Months	Small family owned	Dairy	0	2	0	2	1	2	2	2	0	0
2021	Edirne	2	3-6 Months	Small family owned	Meat	0	4	0	4	2	3	4	3	0	0
2021	Edirne	5	3-6 Months	Small family owned	Dairy	0	1	0	1	0	1	1	1	0	0
2021	İstanbul	10	0-3 Months	Small family owned	Meat	0	4	0	4	0	4	4	4	0	0
2021	İstanbul	10	3-6 Months	Small family owned	Meat	0	1	0	1	0	1	1	1	0	0
2021	İstanbul	10	0-3 Months	Small family owned	Dairy	0	2	0	2	0	2	2	2	0	0
2021	Tekirdağ	11	0-3 Months	Small family owned	N/A	0	1	0	1	1	1	1	1	0	0
2021	Tekirdağ	12	0-3 Months	Small family owned	N/A	0	3	0	3	3	3	3	3	0	0
2021	Tekirdağ	13	0-3 Months	Small family owned	N/A	0	3	0	3	3	3	3	3	0	0
2021	Tekirdağ	14	0-3 Months	Small family owned	N/A	0	2	0	2	2	2	2	2	0	0
2021	Tekirdağ	15	0-3 Months	Small family owned	N/A	0	5	0	5	5	5	5	5	0	0
2021	Tekirdağ	16	3-6 Months	Small family owned	N/A	0	1	0	1	1	1	1	1	0	0
2021	Tekirdağ	17	0-3 Months	Small family owned	N/A	0	2	0	2	2	2	2	2	0	0
2021	Kırklareli	18	0-3 Months	Small family owned	Etçi	0	3	2	1	2	3	3	3	0	1
2021	Kırklareli	19	0-3 Months	Small family owned	N/A	0	6	0	6	2	3	1	1	0	0
2021	Kırklareli	20	0-3 Months	Integrated	Dairy	1	1	0	1	1	1	1	1	0	0
2021	Kırklareli	21	0-3 Months	Integrated	Dairy	4	8	5	3	7	8	8	8	1	5
2021	Kırklareli	21	0-3 Months	Integrated	Meat	0	3	2	1	2	3	3	3	0	2
2021	Kırklareli	22	0-3 Months	Integrated	Dairy	12	16	2	14	15	14	14	14	0	2
2021	Kırklareli	22	0-3 Months	Integrated	Meat	2	5	2	3	4	4	4	4	0	2
2021	Kırklareli	23	0-3 Months	Integrated	Dairy	13	18	16	2	3	3	3	1	0	16
2021	Kırklareli	23	0-3 Months	Integrated	Meat	7	12	7	5	5	4	4	2	0	7
2021	Kırklareli	24	0-3 Months	Integrated	Dairy	1	9	8	1	5	4	6	5	0	8
2021	Kırklareli	24	0-3 Months	Integrated	Meat	0	1	1	0	0	0	0	0	0	1
2021	Kırklareli	25	0-3 Months	Integrated	Dairy	8	22	12	10	14	13	12	9	0	12
2021	Kırklareli	25	0-3 Months	Integrated	Meat	15	34	21	13	21	21	22	18	0	21
2021	Kırklareli	20	3-6 Months	Integrated	Dairy	5	5	0	5	5	4	5	2	0	0
2021	Kırklareli	20	3-6 Months	Integrated	Meat	3	3	0	3	2	2	3	2	0	0
2021	Kırklareli	22	3-6 Months	Integrated	Dairy	2	2	0	2	2	2	2	2	0	0
2021	Kırklareli	23	3-6 Months	Integrated	Dairy	2	2	0	2	2	2	1	0	0	0
2021	Kırklareli	23	3-6 Months	Integrated	Meat	1	1	0	1	1	1	1	0		0
2021	Kırklareli	24	3-6 Months	Integrated	Dairy	4	4	1	3	4	4	4	4	0	1
2021	Kırklareli	25	3-6 Months	Integrated	Dairy	2	2	0	2	2	2	2	2	0	0
2021	Kırklareli	25	3-6 Months	Integrated	Meat	2	2	0	2	2	2	2	2	0	0
2022	Kırklareli	21	0-3 Months	Integrated	Dairy	22	37	36	1	9	7	11	5	3	37
2022	Kırklareli	21	0-3 Months	Integrated	Meat	3	5	5	0	1	1	1	0	1	5
2022	Kırklareli	23	0-3 Months	Integrated	Dairy	14	30	23	7	2	10	9	7	4	23
2022	Kırklareli	23	0-3 Months	Integrated	Meat	1	5	2	3	0	4	3	3	1	2
2022	Kırklareli	21	3-6 Months	Integrated	Dairy	2	2	2	0	1	1	2	1	2	2
2022	Kırklareli	21	3-6 Months	Integrated	Meat	5	5	1	4	5	5	5	4	0	1
2022	Kırklareli	23	3-6 Months	Integrated	Dairy	41	41	34	7	18	19	14	8	2	34
2022	Kırklareli	23	3-6 Months	Integrated	Meat	2	2	2	0	0	2	2	0	0	2
2022	Kırklareli	21	6-12 Months	Integrated	Dairy	12	12	4	8	12	11	9	5	0	4
2022	Kırklareli	21	6-12 Months	Integrated	Meat	5	5	0	5	5	5	5	5	0	0

2022	Kırklareli	23	6-12 Months	Integrated	Dairy	9	9	0	9	8	8	7	6	0	0
2022	Kırklareli	23	6-12 Months	Integrated	Meat	3	3	0	3	3	3	3	3	0	0
2022	Kırklareli	21	1-2 Years	Integrated	Dairy	18	18	8	10	15	15	15	9	0	8
2022	Kırklareli	23	1-2 Years	Integrated	Dairy	12	12	3	9	12	12	11	11	0	3
2022	Kırklareli	21	2-3 Years	Integrated	Dairy	2	2	0	2	2	2	2	2	0	0
2022	Kırklareli	23	2-3 Years	Integrated	Dairy	7	7	4	3	6	6	6	2	1	4
2022	Kırklareli	21	3-4 Years	Integrated	Dairy	6	6	0	6	6	6	5	4	0	0
2022	Kırklareli	23	3-4 Years	Integrated	Dairy	6	6	1	5	6	6	6	6	0	1
2022	Kırklareli	21	4-5 Years	Integrated	Dairy	2	2	0	2	2	2	2	2	0	0
2022	Kırklareli	23	4-5 Years	Integrated	Dairy	2	2	0	2	2	2	2	1	0	0
2022	Kırklareli	23	6-7 Years	Integrated	Dairy	2	2	1	1	2	1	1	1	0	1
2022	Kırklareli	23	9-10 Years	Integrated	Dairy	1	1	0	1	1	1	1	0	0	0
2023	Kırklareli	26	3-6 Months	Small family owned	Meat	0	12	5	7	7	7	7	8	1	5
2023	Kırklareli	21	0-3 Months	Integrated	Dairy	2	2	2	0	0	0	0	0	0	2
2023	Kırklareli	23	0-3 Months	Integrated	Dairy	1	1	1	0	1	1	1	0	0	1
2023	Kırklareli	21	3-6 Months	Integrated	Dairy	17	17	15	2	3	5	4	4	1	15
2023	Kırklareli	21	3-6 Months	Integrated	Meat	4	4	1	3	3	3	3	3	0	1
2023	Kırklareli	23	3-6 Months	Integrated	Dairy	20	20	18	2	9	7	7	4	0	18
2023	Kırklareli	23	3-6 Months	Integrated	Meat	3	3	2	1	1	1	1	1	0	2
2023	Kırklareli	21	6-12 Months	Integrated	Dairy	9	9	7	2	5	4	4	5	0	7
2023	Kırklareli	23	6-12 Months	Integrated	Dairy	4	4	1	3	3	3	3	2	0	1
2023	Kırklareli	21	1-2 Years	Integrated	Dairy	5	7	5	2	6	6	6	4	0	5
2023	Kırklareli	23	1-2 Years	Integrated	Dairy	9	9	6	3	7	8	7	7	0	6
2023	Kırklareli	23	2-3 Years	Integrated	Dairy	2	2	2	0	1	0	0	1	0	2
2023	Kırklareli	21	3-4 Years	Integrated	Dairy	3	3	3	0	2	1	2	1	0	3
2023	Kırklareli	23	3-4 Years	Integrated	Dairy	3	3	1	2	2	3	2	2	0	1
2023	Kırklareli	21	5-6 Years	Integrated	Dairy	2	2	0	2	2	2	2	2	0	0
						**TOTAL**		**274**	**259**						
