# Supplementary material for: Genotypes of Bovine Viral Diarrhea Virus Infecting Vaccinated and Nonvaccinated Cattle in Thrace District, Türkiye
Source: Viruses. 2026 Jul 23;18(8):807. doi: 10.3390/v18080807 (PMC13517928; doi:10.3390/v18080807)
Supplement: Supplementary file 1 [file viruses-18-00807-s001.zip › Supplementary Table S2.pdf]

Supplementary Table S2 - List of reference strains of pestiviruses for phylogenetic comparison with isolates

**Pestivirus species**	**Subtype**	**Strain**	**Origin**	**Location**	**Collection year**	**5'UTR Accession number**
BVDV-1	1a	NADL	Cattle	USA	1963	AJ133739
BVDV-1	1a	Oregon C24 V	Cattle	USA	1998	AF091605.1
BVDV-1	1a	BJ-2013	Cattle	China	2013	MH490942.1
BVDV-1	1a	SWU-Z6	fecal	China	2016	MF693403
BVDV-1	1b	BJ-2016	Cattle	China	2016	MH490943.1
BVDV-1	1b	CP7	Bos	Germany	1996	U63479.1
BVDV-1	1b	AV69	Cattle	USA	2011	KC695814
BVDV-1	1b	3156	Cattle	China	2011	JN704144
BVDV-1	1c	Crookwell	Cattle	Australia	1989	JQ743606.1
BVDV-1	1c	MF-1	Cattle	China	2016	KY865369
BVDV-1	1c	Bega-like	Bovine	Australia	2012	KF896608
BVDV-1	1c	NM2103	Cattle	China	2021	ON337882
BVDV-1	1d	10-JJ-SKR	Cattle	South Korea	2010	KC757383
BVDV-1	1d	SWU-DJ2	yak	China	2015	MF166858
BVDV-1	1d	F	-	-	-	AF298065.1
BVDV-1	1e	68-1	Cattle	UK	2008	MW250802
BVDV-1	1e	S03-1175	Cattle	USA	2003	MW655631
BVDV-1	1e	SLO/2407/2006	Cattle	Slovenia	2006	KX577637
BVDV-1	1f	192-KW/17	Cattle	Poland	2019	MK381368.1
BVDV-1	1f	210-GK/18	Cattle	Poland	2018	MK381386
BVDV-1	1g	48/08	Cattle	Poland	2008	JN715004.1
BVDV-1	1g	10/08	Cattle	Poland	2008	JN715004.1
BVDV-1	1h	BG9a02	Bovine	Italy	2002	MG434576.1
BVDV-1	1h	CR1a99	Bovine	Italy	1999	MG434575
BVDV-1	1i	ACM/BR/2016	Cattle	Brazil	2017	KX857724
BVDV-1	1i	CA2006	Bovine	USA	2006	MK775204
BVDV-1	1j	A469	Bovine	Chile	2014	GU987129.1
BVDV-1	1j	2/Vr/95	Bovine	Italy	2000	AJ293594.1
BVDV-1	1j	KS 86-1 ncp	Cattle	Japan	2002	AB078950
BVDV-1	1k	R3230-95	Cattle	Switzerland	1995	MW655628
BVDV-1	1k	TO/197/11	Cattle	Italy	2011	MW054935
BVDV-1	1L	71-15	Bos taurus	France	2005	KF205306
BVDV-1	1L	71-16	Bos taurus	France	2008	KF205307.1
BVDV-1	1m	SD-15	Cattle	China	2015	KR866116
BVDV-1	1m	CHN/HB-01/2017	bovine	China	2017	ON901784
BVDV-1	1n	Shitara/02/06	Cattle	Japan	2006	LC089876
BVDV-1	1o	HY-3	Cattle	China	2016	KY865366.1
BVDV-1	1o	BVDV-NM2312	Cattle	China	2023	PP992316
BVDV-1	1p	TJ06	Cattle	China	2006	GU120246.1
BVDV-1	1p	BJ0702	Cattle	China	2007	GU120248.1
BVDV-1	1p	BJ0703	Cattle	China	2007	GU120249.1
BVDV-1	1q	Camel-6	Camel	China	2010	KC695810
BVDV-1	1q	SD0803	pig	China	2008	JN400273
BVDV-1	1r	VE/245/12	-	Italy	2014	LM994671
BVDV-1	1r	CA/181/10	-	Italy	2015	LM994672
BVDV-1	1t	SI/207/12	-	Italy	2014	LM994674
BVDV-1	1v	EN-6	Cattle	China	2017	MN417813.1

BVDV-1	1v	HB-03	Cattle	China	2018	ON901785.1
BVDV-1	1v	GA190608	Cattle	China	2019	MT933204.1
BVDV-2	2a	HLJ-10	Cattle	China	2011	JF714967
BVDV-2	2b	SD1301	Cattle	China	2012	KJ000672
BVDV-2	2c	SH2210-23	Cattle	Germany	2010	HG426494
BVDV-3	3	XJ-BVDV-3	bovine	China	2022	OP210314.1
BVDV-3	3	Th/04 KhonKaen	bovine	Thailand	2004	NC_012812
CSFV	CSFV	Shimen	swine	China	1999	AF092448.2
CSFV	CSFV	SXCDK	swine	China	2009	GQ923951
BDV	BDV	JSLS12-01	Ovis aries	China	2012	KC963426.1
BDV	BDV	X818	Sheep	Germany	1997	NC_003679.1
