# Supplementary material for: Genotypes of Bovine Viral Diarrhea Virus Infecting Vaccinated and Nonvaccinated Cattle in Thrace District, Türkiye
Source: Viruses. 2026 Jul 23;18(8):807. doi: 10.3390/v18080807 (PMC13517928; doi:10.3390/v18080807)
Supplement: Supplementary file 1 [file viruses-18-00807-s001.zip › Supplementary Table S3.pdf]

Supplementary Table S3. Sample identity, sample type, sampling year, sampled city, BVDV genotype detected, Ct values of positive samples, age and vaccination status of animals and GenBank accession numbers of BVDV sequences obtained in this study.

**Sample ID**	**Sample type**	**Sampling year**	**City**	**BVDV Genotype/Subgenotype**	**Ct values**	**animal age**	**Vaccination status**	**GenBank accession numbers**
2115	Nasal swab	2022	Kırklareli	*Pestivirus bovis* subgenotype 1a	26.06	1-2 years	Yes	PQ432880
3937	Nasal swab	2022	Kırklareli	*Pestivirus bovis* subgenotype 1a	26.7	3-4 years	Yes	PQ432881
6924	Nasal swab	2023	Kırklareli	*Pestivirus bovis* subgenotype 1a	22.05	3-6 months	No	PQ432875
6949	Nasal swab	2023	Kırklareli	*Pestivirus bovis* subgenotype 1a	22.27	3-6 months	No	PQ432877
7043	Nasal swab	2023	Kırklareli	*Pestivirus bovis* subgenotype 1a	25.03	3-6 months	No	PQ432876
7453	Nasal swab	2023	Kırklareli	*Pestivirus bovis* subgenotype 1a	22.55	3-6 months	No	PQ432878
7715	Nasal swab	2023	Kırklareli	*Pestivirus bovis* subgenotype 1a	25.25	3-6 months	No	PQ432873
7792	Nasal swab	2023	Kırklareli	*Pestivirus bovis* subgenotype 1a	24.85	3-6 months	No	PQ432879
7817	Nasal swab	2023	Kırklareli	*Pestivirus bovis* subgenotype 1a	25.35	3-6 months	No	PQ432874
1002	Nasal swab	2021	Edirne	*Pestivirus bovis* subgenotype 1f	17.66	3-6 months	No	PQ432882
2711	Rectal swab	2023	Kırklareli	*Pestivirus brazilense*	27.85	3-6 moths	No	PQ432872
